# Optimization of Epoxy Resin: An Investigation of Eggshell as a Synergic Filler

**DOI:** 10.3390/ma12091489

**Published:** 2019-05-08

**Authors:** José William de Lima Souza, Nichollas Guimarães Jaques, Matthias Popp, Jana Kolbe, Marcus Vinícius Lia Fook, Renate Maria Ramos Wellen

**Affiliations:** 1 Academic Unit of Materials Engineering, Federal University of Campina Grande, Campina Grande 58249-140, Brazil; william.souza@certbio.ufcg.edu.br (J.W.d.L.S.); nichollasemat@hotmail.com (N.G.J.); marcus.liafook@certbio.ufcg.edu.br (M.V.L.F.); 2 Materials Engineering Department, Federal University of Paraiba, João Pessoa 58051-900, Brazil; 3 Fraunhofer-Institut für Fertigungstechnik und Angewandte Materialforschung (IFAM), Wiener Straße 12, D-28359 Bremen, Germany; matthias.popp@ifam.fraunhofer.de (M.P.); jana.kolbe@ifam.fraunhofer.de (J.K.)

**Keywords:** epoxy, eggshell, cure kinetics, mechanical properties, morphology

## Abstract

Epoxy resin based on bisphenol A diglycidyl ether/anhydride methyl tetrahydrophthalic/2,4,6-tris(dimethylaminomethyl)phenol (DGEBA/MTHPA/DEH 35) was produced by magnetic stirring; chicken eggshell (ES) was added as cure improver. Thermal stability, cure parameters, mechanical properties, and fracture surface were investigated by thermogravimetry (TGA), differential scanning calorimetry (DSC), tensile experiments, and scanning electron microscopy (SEM). In general, the addition of ES slightly decreased the thermal stability, being T_0.05_ 5% lower than that of the reference sample. The cure rate increased with the heating rates, while best results were obtained upon addition of neat membrane (M) from ES. Surprisingly, the mechanical properties were significantly improved with ES as well as with M, being the Young’s modulus 18% higher, the tensile strength 50% higher, and the deformation 35% higher than those of epoxy resin. SEM images showed that the synthetic compounds presented a smooth fracture surface, while the compounds with ES and M had a rougher surface with multiplane fractures, suggesting a fracture with higher energy absorption. In conclusion, epoxy/ES composites with better performance were produced, and effective tools are provided to control and attain in the future even better properties with ecological features.

## 1. Introduction

Epoxy is the thermosetting polymer most widely used in the industry, mainly in electronics and aerospace, due to its characteristics such as adhesiveness, low shrinkage, high strength, excellent electrical insulation, and corrosion resistance [1,2]. The most common epoxy resins are the rigid aromatic ones made from bisphenol A diglycidyl ether (DGEBA), such as D.E.R. 331, which is the most largely produced epoxy resin for industrial sites. The tough, insoluble, and infusible epoxy is normally formed via a cross-linking reaction (also called the curing process or solidification) of liquid epoxy resins with hardeners (also called catalysts or curing agents) including polyfunctional amines, acids (or acid anhydrides), phenols, alcohols, and thiols. Nevertheless, brittleness and low impact strength [2,3] are drawbacks that prevent wider applications. Aiming at the improvement of these properties, reinforcements have been incorporated; however, high costs, difficulty of production, and the propensity to agglomeration prevent them from being unattractive products. In addition, the growing concern over sustainability and the environmental impact of petroleum-based polymer materials promotes the utilization of renewable feedstock for industrial materials, which appear promising because of its low cost and minimal environment impact [3,4,5].

Within different kinds of biowaste additives, the widely available inexpensive chicken eggshell (ES) is a promising candidate as a reinforcing agent in polymeric industries, because of its good mechanical characteristics such as acceptable toughness and impact strength [4,5]. In Brazil, egg production generates annually about 16,521 tons of waste [6] and is assumed to be one of the food industry branches of highest environmental impact. ES contains 95% CaCO_3_ (calcium carbonate) and 5% of organic components such as proteins, collagen, and polysaccharides [7], and can be a favorable thermosetting reinforcement in the presence of nanopores on its surface, which may improve the interfacial interaction between filler and matrix. Furthermore, amine and carboxylic functional groups present in proteins could benefit the cure process. Compared to pristine CaCO_3_, ES can more largely contribute with cross-linking reactions, as verified by Saeb et al. during isothermal kinetics analyses. ES also favors the formation of the hydrogen bonds which may drive the formation of higher performance composites [4,5,8,9,10,11,12,13,14,15,16].

It is well established that the ultimate properties (as thermal, mechanical, and rheological, for instance) of thermosetting materials are highly dictated by their curing characteristics, allowing one to control the processing parameters. Moreover, the study of the cure kinetics of filled epoxy provides quantitative information regarding the effects of additives on microstructural features of 3D cured resin. Parameters that can be optimized for the best cure performance are the amount and reactivity of reagents as well as the time and temperature of the curing process [1,4,5,17,18].

Several hardeners, catalyzers and fillers are used for epoxy curing. Deka et al. [19] reported the curing reaction of epoxy/distillers dried grain from corn by non-isothermal DSC analysis; it was observed that the filler reduced the curing temperature, and, additionally, the process was faster than in neat epoxy. It was suggested the filler acted as cross-linking improver, overcoming the decrease in the cure rate caused by steric hindrances. Hsieh et al. [20] investigated the curing behavior of epoxy/silica–carbon inorganic fillers from rice husk ash. The cure of neat epoxy was characterized by a double exothermic peak, while the composites presented a single peak which showed a reduction in the temperature and enthalpy upon increase of the filler content, indicative of a modification of the mechanism modifier and an acceleration of the reaction. Zieleniewska et al. [21] evaluated the effects of eggshell waste as filler in rigid polyurethane. They observed a rearrangement of the rigid segments related to the flexible ones caused by the introduction of ES, which resulted in a rise of phase separation that led to an increased physical cross-linking of the composites, providing higher thermal stability, increased apparent density, no toxicity, and resistance towards bacteria adhesion. Saeb et al. [5] performed a comparative study of epoxy curing kinetics upon addition of ES and CaCO_3_. The fillers were superficially applied, and their effect evaluated. ES without modification at a concentration of 0.1% presented the lowest values of activation energy and cure temperature. The authors alleged these data were the result of peptidic groups present in ES providing a better interaction with the polar groups of epoxy, thus minimizing the steric hindrance.

Although the study of epoxy with ES has already started [4,5], in this work, ES was added as an additive and filler, presenting a novel approach to substitute synthetic catalyzers with a natural one and providing an alternative to food waste reuse and cost reduction. Therefore, this work aims to study the cure kinetics of epoxy/ES composites through differential scanning calorimetry (DSC), to investigate ES effects on mechanical behavior, and to investigate fracture mechanisms through scanning electron microscopy (SEM).

## 2. Experimental

### 2.1. Materials

Bisphenol A diglycidyl ether (DER 331) with epoxide equivalent weight of 182–192 g/eq, anhydride methyl tetrahydrophthalic (MTHPA) and 2,4,6-tris(dimethylaminomethyl)phenol (DEH 35) were supplied by Olin Corporation (São Paulo, Brazil). Chicken eggshell was supplied by a local farm (Campina Grande-PB, Brazil). 

### 2.2. Methods

#### 2.2.1. Eggshell Powder Processing

Processing of eggshell powder and membrane was performed as an adaptation of the methodology proposed by Santos et al. [22]. ES was washed in sodium hypochlorite (NaClO) and afterwards immersed in water for 2 h to remove the membrane. ES and membrane were dried in oven at 100 °C for 24 h. Both materials were ground in a coffee mill B55 Botini (Bilac, Brazil) and sieved through #325 and #100 mesh, respectively.

#### 2.2.2. Compounding

Epoxy compounding at 100:87 (DER 331/MTHPA/resin/hardener) with DEH 35 at concentrations 0, 1, 2, and 5 pcr (parts per hundred) were mixed in a magnetic stirrer for 5 min at 800 rpm.

ES at contents of 0.5, 5, and 10 pcr was added into 100:87 (DER 331/MTHPA). Afterwards, these compounds were mixed in a magnetic stirrer from Corning (Reynosa, Mexico) for 5 min at 800 rpm at ambient temperature (~23 °C), and then the filler (ES) was dispersed by sonication for 2 min at ~23 °C. Compounds produced in this work are coded as presented in Table 1. 

On the basis of the thermal properties determined from DSC analysis, specimens of selected compounds were produced according to ASTM D638-14 standard and by applying thermal profiles as presented in Table 2. Their mechanical behavior and morphological characters were investigated.

#### 2.2.3. Characterization of Epoxy Compound

Thermal stability was investigated by thermogravimetry (TGA). The tests were carried out in TGA Pyris-1 from Perkin Elmer (Waltham, MA, USA). Samples of approximately 5 mg were heated from 30 to 900 °C at a heating rate of 10 °C/min under synthetic air with gas flow of 20 mL/min.

The curing process was analyzed by differential scanning calorimetry (DSC), using a DSC Q20 from TA Instruments (New Castle, DE, USA). Samples of approximately 5 mg were tested in a standard closed aluminum pan, under a nitrogen gas flow 50 of mL/min. The samples were heated from 30 °C to 400 °C, at heating rates of 1, 2, 5, 10, and 20 °C/min.

Tensile tests were performed in triplicate using a universal test machine, Instron 3366 (Norwood, MA, USA), according to ASTM D638-14 at ambient temperature (~23 °C) and deformation rate of 5 mm/min.

The fracture surface and filler dispersion of the epoxy/anhydride/catalyzer system were analyzed using a scanning electron microscope (SEM) from World Phenom Pro X800-08334 (Eindhoven, The Netherlands).

## 3. Results and Discussion

### 3.1. Evaluation of Thermal Stability by TGA

**Figure 1:**
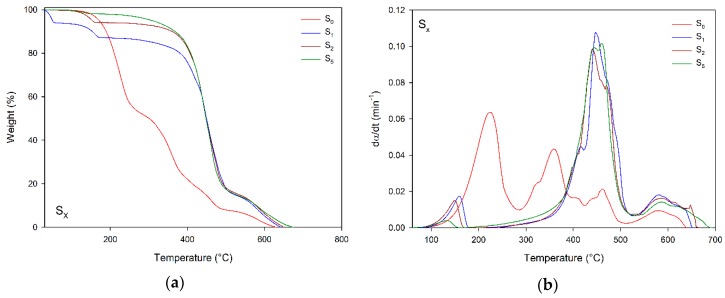
(**a**) TGA plots and (**b**) decomposition rate (dα/dt) of Synthetic (S_X_) compounds—Benchmark.

Figure 1 presents TGA plots of S_x_ compounds and their decomposition rates. The benchmark S_0_ 100:87:0 system has four decomposition steps, which range from 78 to 287 °C, from 287 to 425 °C, from 425 to 512 °C, and from 512 to 639 °C. It is worth of mention that the curing took place during heating, as also the decomposition. According to Montserrat et al. [23], in the absence of a catalyzer, the thermal degradation of DGEBA with anhydride as a hardener begins at temperatures higher than 300 °C. Therefore, the first and second weight loss suggest the decomposition of unreacted hardener (MTHPA) and non-cross-linked epoxy (additional details may be checked in Supplementary Materials). The third and fourth steps are related to the decomposition of cross-linked epoxy and carbon residue generating two products of degradation, CO_2_ and H_2_O [23,24,25].

Upon addition of DEH 35, three decomposition steps occurred verified. According to Meadows et al. [26], during DER 331 curing with MTHPA and DEH 35, hydroxyl is released as a by-product, which is volatilized in the first step of decomposition, ranging from 76 to 272 °C for 5 parts of DEH 35. The second and third steps, which range from 164 to 529 °C and from 529 to 689 °C for 5 parts of DEH 35, are related to the decomposition of epoxy releasing CO_2_ and H_2_O [23,24,25]. In contrast to the S_0_ system, compounds with DEH 35 had no decomposition associated with non-cross-linked epoxy; in fact, the increase of DEH 35 resulted in higher weight loss in the third step, suggesting the amount of catalyst significantly influenced epoxy cross-link.

**Figure 2:**
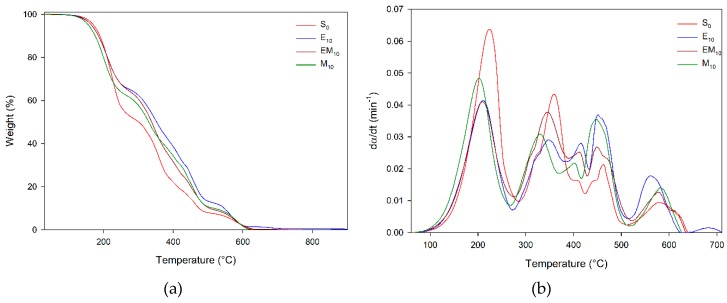
(**a**) TGA plots and (**b**) decomposition rate (dα/dt) of bio-based compounds.

The TGA plots of epoxy/eggshell systems (E_10_, EM_10_, and M_10_) presented a similar thermal behavior to that of benchmark S_0_, as shown in Figure 2. For these systems, four decomposition steps suggested the decomposition of unreacted hardener, non-cross-linked epoxy, cross-linked epoxy, and carbon residue [23,24,25]. From individual analyses of the decompositions steps of S_0_, E_10_, EM_10_, and M_10_, in the first step of S_0_ there was an increase of approximately 10% in weight loss of MTHPA in comparison with the epoxy/eggshell system, suggesting that a smaller amount of hardener had reacted in the curing process. The decomposition in the second and third steps was competitive, since one was associated with the decomposition of the non-cross-linked epoxy, and the other with the decomposition of the cross-linked portion. Thus, M_10_ presented a more pronounced weight loss in the third stage, suggesting this system had a greater fraction of reticulation, i.e., was more effectively cured.

Table 3 presents thermogravimetric parameters of epoxy compounds, additionally to weight loss data, onset (T_0_), and final (T_f_) decomposition temperatures, as well as decomposition rates. The thermal stability of epoxy was evaluated by the parameter τ_1/2_—time at the 50% decomposition conversion degree—which increased with higher amounts of DEH 35, suggesting the catalyzer improved the thermal stability of epoxy. In regard to the epoxy/eggshell compounds, E_10_ presented a higher value for τ_1/2_ than EM_10_ and M_10_. Nevertheless, the S_5_ compounds showed an increase of 29% (10.7 min) in τ_1/2_ in comparison with E_10_, indicating that a better cross-link reaction—present in S_x_ compounds—produced an enhanced thermal decomposition.

### 3.2. DSC Measurements

**Figure 3:**
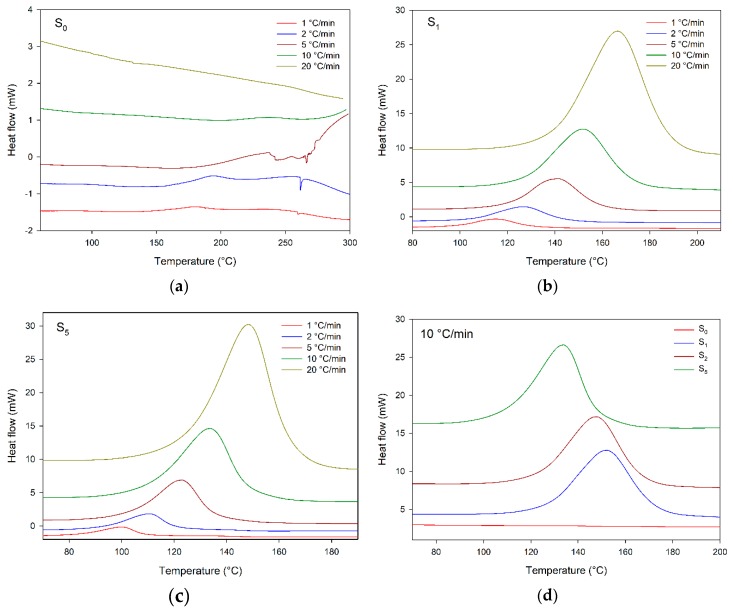
DSC scans of S_X_ benchmark compounds (**a**) S_1_, (**b**) S_2_, (**c**) S_5_, and (**d**) S_0_, S_1_, S_2_, and S_5_ computed at 10 °C/min.

Figure 3 shows DSC scans of S_X_ compounds. The uncatalyzed system S_0_ does not have a complete exothermic peak in the analyzed temperature range for heating rates higher than 2 °C/min. This corresponds to the results of the TGA curves, i.e., the cure process without catalyzer occurred at temperatures higher than 300 °C at the applied heating rates [23,24,25].

Upon addition of DEH 35 catalyzer, an exothermic peak appeared, which ranged from 94 to 175, 90 to 186, 78 to 158, for 1, 2, and 5 parts of DEH 35 at 5 °C/min (temperatures and cure parameters associated with the exothermic peaks are displayed in Table 4). The exothermic peak presenting a bell shape suggests curing taking place in one reaction model, whereas no discontinuities were observed [27,28,29]. These peaks were displaced to higher temperatures, increasing the heating rates. Regarding DEH 35 addition, the peaks appeared at lower temperatures upon its increase, evidencing a faster curing.

Ručigaj et al. [30] also observed a similar influence of the catalyzer content and heating rate during investigation of the cure kinetics of an epoxided soy bean oil (ESO)/anhydride/triazole system, in which the presence of a catalyzer displaced the onset temperature of the exothermic peak to ~120 °C in comparison with the pure epoxy (240 °C); in contrast, the heating rates shifted the exothermic peak to higher temperatures.

The integration of exothermic peaks presented in Figure 3 provides information related to the cure, i.e., the maximum cure rate (c_max_) and peak temperatures (T_0.01_, T_p_, T_0.999_), which are shown in Table 4.

**Figure 4:**
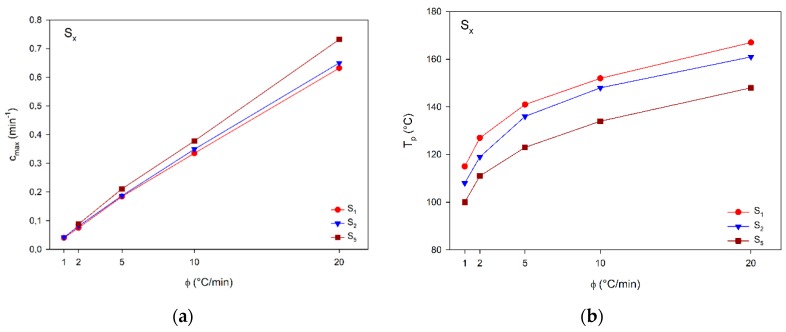
Effect of heating rates and DEH 35 content on (**a**) c_max_ (min^−1^) and (**b**) T_p_ (°C). Benchmark compounds.

Figure 4a presents the effect of heating rates on c_max_ of S_x_ compounds. This parameter can be understood as the reaction speed. Looking at Figure 4a, a linear trend is observed, i.e., the cure was faster for higher heating rates, and this increase was greater for S_5_ compound which presented c_max_ of 0.688 min^−1^, higher than those of other compounds.

Regarding the influence of DEH 35 on c_max_, increasing its content provided higher c_max_, and this trend was more pronounced for higher heating rates; for instance, at 20 °C/min, c_max_ of S_5_ was 16% higher than those of other compounds with lower content of DEH 35, suggesting the catalyzer addition accelerated the cure.

Concerning the effect of heating rates and DEH 35 content on T_p_ shown in Figure 4b, at higher heating rates, T_p_ assumed higher values, while, after the addition of DEH 35, T_p_ decreased, i.e., the cure took place at lower temperatures. Therefore, both heating rate and DEH 35 content can be used as safe tools to control the curing as required by the industrial process.

**Figure 5:**
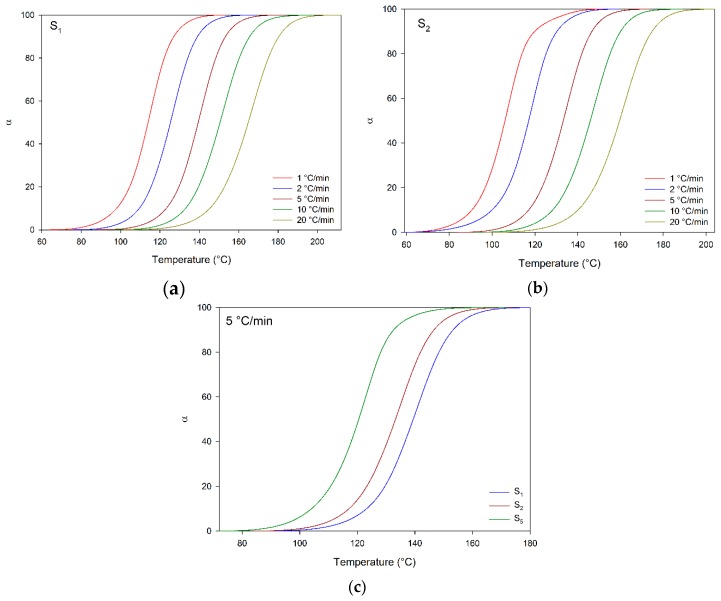
Cure conversion of benchmark compounds (S_x_). (**a**) S_1_, (**b**) S_2_, (**c**) S_1_, S_2_, and S_5_ computed at 5 °C/min.

Figure 5 shows plots of cure conversion (α) versus temperature for S_X_ compounds; these data were computed from DSC scans. All plots presented a sigmoidal shape without discontinuities, suggesting the cure progressed as an autocatalytic reaction [27,31,32], agreeing with the DSC scans indicating that it took place according to a single “reaction model” as indicated by the observed single peaks. In general, the “S” plots may be divided in three stages: initially, around 0 to 5% of process conversion, they have a slow rate probably due to catalyzation reactions/production of active centers; during the second step—around 5 to 90%—the cure quickly increases, as a result of the availability of functional groups and easy molecular movement; afterwards, the cure proceeds with a delayed rate, because of competitiveness between high viscosity and decreased presence of functional groups. Finally, the viscosity significantly increases, and there is a decrease of reactive groups near the cure end [27,29,32]. Regarding the effect of the heating rates, the “S” plots corresponded to higher temperatures using higher heating rates; this effect was associated with the time dependence of the cure reaction, showing an opposite trend compared to that observed upon DEH 35 addition, which had an accelerator influence over the cure according to the DSC curves.

**Figure 6:**
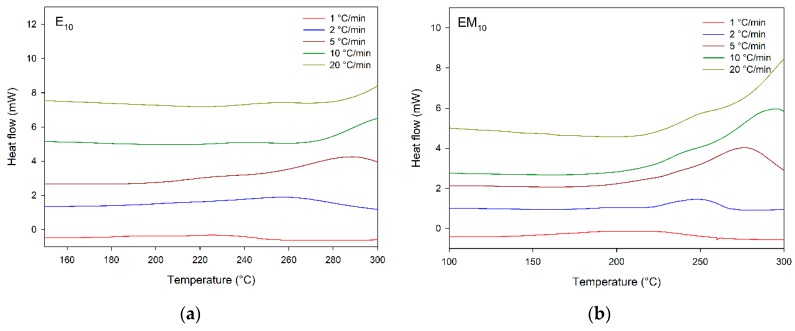

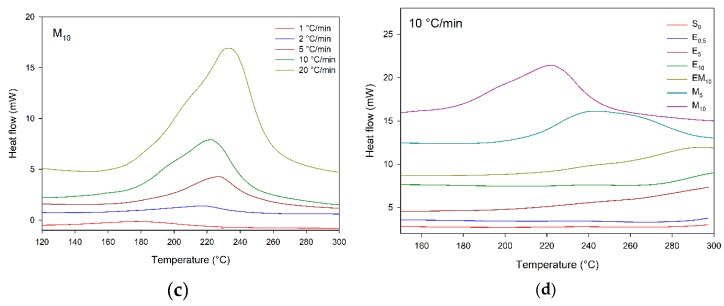
DSC scans of E_X_/EM_X_/M_X_ (**a**) E_10_, (**b**) EM_10_, (**c**) M_10_, and (**d**) DSC acquired at 10 °C/min, compounds indicated.

Figure 6 illustrates the DSC scans of E_x_/EM_x_/M_x_. The compounds containing the membrane displayed exothermic peaks at all heating rates applied in the investigated temperature ranges. This positive response was probably linked to the fact that the membrane contains higher amounts of reactive functional groups such as sulfur constituents than other parts of the chicken eggshell [7]. Thus, the membrane is the best structure for catalyzing the cure reaction of DER 331. Besides that, these data justify the higher weight loss in the third step, related to the cross-linked decomposition content of M_10_, as seen in the TGA plots (Figure 2), confirming the best cure process in this system. As already verified in the S_x_ system, M_x_ presented an exothermic peak with a bell shape, suggesting a single cure reaction model without discontinuities. The cure peaks appeared at higher temperatures with increasing heating rates; as presented above for S_x_ compounds, the thermal transitions depended on the process time. Additionally, when increasing the membrane content, the cure occurred at lower temperatures, suggesting the membrane contains more reactive functional groups in its structure.

The temperature ranges and cure parameters of M_x_ are presented in Table 5, as well as the influence of heating rates and M content on the cure parameters c_max_, T_p_, and overall reaction heat (ΔH). Following a similar trend as observed for S_x_ compounds (S_1_, S_2_, and S_5_), increasing the heating rates resulted in a higher value of c_max_, being more significant for M_10_, with a difference of 0.407 min^−1^ (487%). Among the investigated M contents, for all the heating rates used, M_10_ presented a gain of 91% (0.167 min^−1^). Concerning the influence of the heating rates and M amount on T_p_, the increase of the heating rate resulted in higher T_p_; on the other hand, M_10_ had lower T_p_ than M_5_. Regarding the effect of the heating rates and filler addition on ΔH, interesting trends were observed, as shown in Table 4, i.e., for the parameters measured for synthetic composites, there was a decrease in ΔH when increasing the heating rates, suggesting a lack of time for full polymerization (all reactive groups) leading to lower ΔH values (energy related to exotherm reaction). On the other hand, according to Table 5, for composites containing the membrane (M), ΔH increased with the heating rates: in this situation, functional groups of M exerted a synergic effect, leading to higher rates and higher degree of polymerization.

The improvement of the cure reaction upon filler addition in the epoxy matrix was investigated by Shanmugharaj and Ryu, [28] who studied the influence of the cure characteristics in epoxy/pristine and superficially modified montmorillonite. These authors verified a displacement of the temperature peak to lower temperatures with montmorillonite addition, as also an increasing of ΔH, indicating a better cure characteristic. Saad et al. [33] evaluated the cure parameters of epoxy containing barium ferrite/polyaniline (PANI) fillers; both DSC scans and cure conversion plots were shifted to lower temperatures when increasing the filler content; the authors suggested this was due to the epoxy group opening by the amine present in the PANI structure.

### 3.3. Mechanical Behavior of Epoxy Compounds

**Figure 7:**
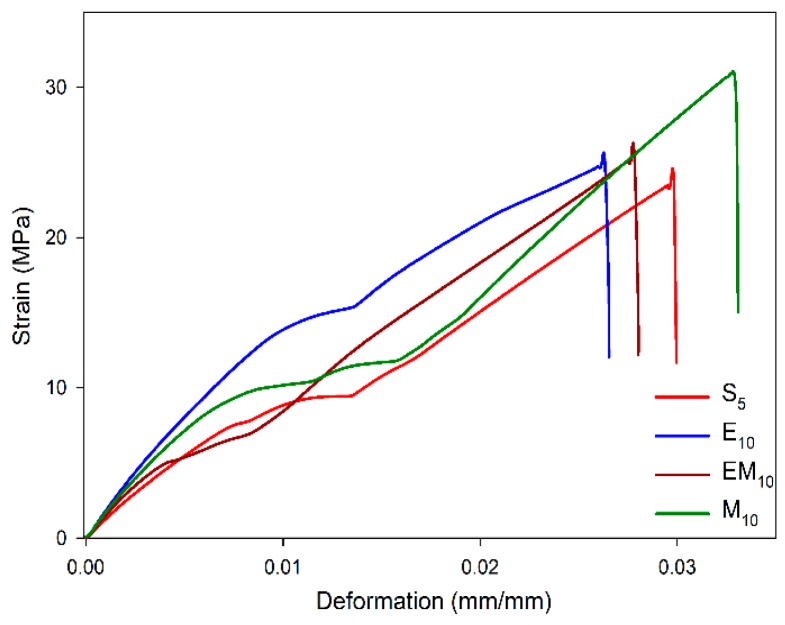
Strain-vs-deformation curves of neat epoxy and epoxy/eggshell compounds.

The tensile properties Young’s modulus, tensile strength, and maximum deformation were evaluated for S_5_, E_10_, EM_10_, M_10_ compounds. The data are presented in Table 6 and Figure 7. The effect of ES and M were clearly identified: the compounds with these fillers displayed higher tensile properties.

E_10_ and EM_10_ presented higher Young’s modulus than S_5_ and M_10_, with an increase of 18% (0.23 GPa) in comparison with the neat epoxy. This effect was associated with the higher stiffness of the CaCO_3_ particles from the eggshell which are able to absorb higher stress with reduced deformation. Meanwhile, M_10_ had the lowest Young’s modulus, nevertheless, it displayed a higher tensile strength and deformation with increases of 47% (8.31 MPa) and 35% (0.82 GPa), respectively. This character could be associated with a better interaction between particle and matrix, as a result of the chemical bonds between the membrane and epoxy established in the cure reaction, which, therefore, can be translated in a good stress transfer [34].

The influence of the fillers on the mechanic behavior of epoxy compounds was investigated by Saeb et al. [34] in epoxy/multiwalled carbon nanotubes (MWCNT). They also analyzed the effect of different surfactants, i.e., polyoxyethylene octyl phenyl ether (Triton X-100), sodium dodecyl sulfate (SDS), and hexadecyl-trimethyl-ammonium bromide (CTAB). According to them, the compounds without surfactant presented a tensile strength reduction of 7.3% (3.3 MPa); on the other hand, the presence of anionic surfactant (SDS) caused an increase of 10.3% (4.7 MPa), while the epoxy/MWCNT system presented higher Young’s modulus than the neat epoxy. Kim et al. [35] reported the influence of graphene oxide filler size on the tensile properties of epoxy/diethytolenediamine; the composites containing graphene oxide showed an improvement in both tensile strength and Young’s modulus, which was greater with larger fillers, leading to 47.57 MPa (99%) and 0.91 GPa (33.2%), respectively.

The great advantages of ES compounds produced in this work depend on the fact that they are bio-based and ecofriendly composites, since eggshell waste was used, and higher thermal as well as mechanical properties were achieved.

### 3.4. Fracture Surface Analysis by SEM

**Figure 8:**
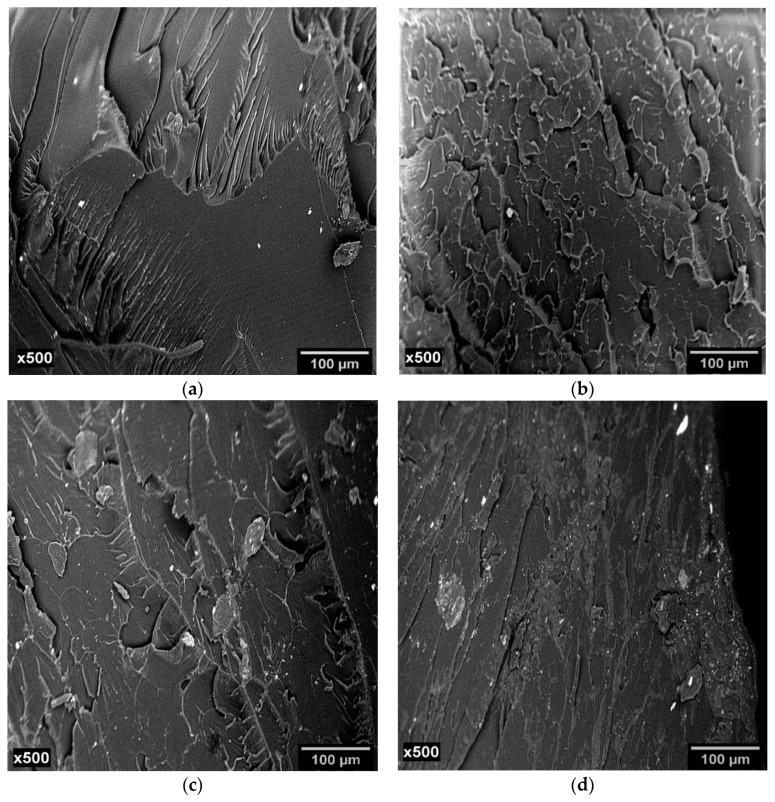
SEM images of fracture surfaces of (**a**) S_5_, (**b**) E_10_, (**c**) EM_10_, and (**d**) M_10_.

Figure 8 shows the SEM images captured from the fracture surface of S_5_, E_10_, EM_10_, and M_10_. The SEM image of S_5_ displays a smooth fracture surface, characteristic of brittle materials with fast failure propagation and low energy absorption. The crack nucleation and its propagation follow a linear direction path, commonly observed with brittle thermosets as epoxy.

In contrast, E_10_, EM_10_, and M_10_—Figure 8b–d presented rougher and multiplane fracture paths, with deviation of the fracture course caused by the presence of eggshell particles which acted as a barrier for the crack propagation. This pattern suggests higher energy absorption during specimen fracture, corroborating the tensile data presented above (Figure 7 and Table 6).

A similar fracture pattern was observed by Saeb et al. [34] in epoxy/MWCNT nanocomposites. The authors observed a flat fracture surface in the neat epoxy; on the other hand, the epoxy/MWCNT/Triton X-100 composite—which had a better particle dispersion—presented a rougher surface than that observed when SDS and CTAB surfactant were present in the epoxy/MWCNT systems, proving that the filler dispersion influenced the fracture mechanism. Zieleniewska et al. [21] studied epoxy/alumoxane and epoxy/bohemite nanocomposite and classified the fracture surface of neat epoxy in three zones: in the first zone, crack propagation was slow, and the surface had was smooth; in the second zone, there was a transition from smooth surface to rough surface, as a result of the increase of crack formation speed; finally, in the third zone, the cracks reached a speed limit, originating new cracks and forming a rougher surface. For the nanocomposite system, the authors observed a high dispersion for the alumoxane system than for the epoxy/bohemite system. Vu and Choi [36] studied an epoxy/microfibril cellulose composite and found that the neat epoxy exhibited a mirror-like surface; however, the fracture surface of the composite was rough, because of the crack deviation inflicted by the filler which acted as a barrier.

## 4. Conclusions

Epoxy composites with addition of chicken eggshell, chicken eggshell plus membrane, and membrane powders were successfully produced in this work, and optimized dispersion was reached as a result of well-designed processing parameters, as observed by SEM images. The thermal stability of eggshell composites was evaluated using TGA, and benchmark and eggshell systems (E, EM, and M) presented a similar behavior. According to DSC data, in general, higher heating rates led to lower ΔH values, while in M composites, a different trend was observed, mostly due to functional groups of M which provided a synergic effect, leading to higher rates and higher degrees of polymerization. The great advantages of eggshell epoxy composites lie on the fact that they are bio-based and ecofriendly composites, since eggshell waste was used, and higher thermal as well as mechanical properties were achieved. Additionally, this work provides safe parameters to control thermal stability as well as cure rate. This paper suggests a large range of topics for new researches, i.e., the use of different epoxy resins, degradation studies, as well as kinetic ones applying theoretical models, with the goal of producing cleaner systems.

## Figures and Tables

**Table 1:** Epoxy compounds and their component contents.

* Composition	Epoxy Resin	Hardener	Catalyzer			
	DER 331	MTHPA	DEH 35	Chicken Eggshell	Chicken Eggshell + Membrane	Membrane
S_0_	100	87	0	0	0	0
S_1_	100	87	1	0	0	0
S_2_	100	87	2	0	0	0
S_5_	100	87	5	0	0	0
E_0.5_	100	87	0	0.5	0	0
E_5_	100	87	0	5	0	0
E_10_	100	87	0	10	0	0
EM_10_	100	87	0	0	10	0
M_5_	100	87	0	0	0	5
M_10_	100	87	0	0	0	10

* S: synthetic compounds. E: composites with eggshell powder. EM: composites with eggshell and membrane powder. M: composites with membrane powder.

**Table 2:** Cure temperature profiles of S_5_, E_10_, EM_10_, and M_10_ compounds.

Time (h)	Temperature (°C)
2	80
4	120
2	140
4	160
2	170
4	190
2	200

**Table 3:** Thermalgravimetric parameters of epoxy compounds.

Composition		S_0_	S_1_	S_2_	S_5_	E_10_	EM_10_	M_10_
First step	T_0_^1^ (°C)	78	73	71	76	66	66	70
	T_f_^2^ (°C)	287	200	187	164	276	285	160
	Weight Loss (%)	48.40	12.68	5.95	1.91	34.72	35.85	38.00
	d_max_^3^ (min^−1^)	0.064	0.017	0.015	0.004	0.041	0.041	0.048
Second step	T_0_ (°C)	287	200	187	164	276	285	286
	T_f_ (°C)	425	527	530	529	390	417	529
	Weight Loss (%)	33.14	72.42	78.77	83.39	35.15	39.37	31.48
	d_max_ (min^−1^)	0.043	0.108	0.099	0.099	0.029	0.038	0.031
Third step	T_0_ (°C)	425	527	530	529	390	417	529
	T_f_ (°C)	512	642	665	689	524	516	691
	Weight Loss (%)	10.82	14.61	15.28	14.71	17.64	15.79	20.63
	d_max_ (min^−1^)	0.021	0.018	0.016	0.014	0.037	0.025	0.036
Fourth step	T_0_ (°C)	512	-	-	-	514	524	516
	T_f_ (°C)	639	-	-	-	632	640	626
	Weight Loss (%)	7.63	-	-	-	11.18	9.00	10.02
	d_max_ (min^−1^)	0.009	-	-	-	0.018	0.013	0.014
Fifth step	T_0_ (°C)	-	-	-	-	632	-	-
	T_f_ (°C)	-	-	-	-	717	-	-
	Weight Loss (%)	-	-	-	-	1.04	-	-
	d_max_ (min^−1^)	-	-	-	-	0.002	-	-
T_0.05_^4^ (°C)		172	164	153	308	164	165	186
T_max_^5^ (°C)		639	642	665	678	717	628	626
τ_1/2_^6^ (min)		21.1	36.0	36.8	36.9	26.2	25.5	24.2

^1^ T_0_ initial decomposition temperature. ^2^ T_f_ final decomposition temperature. ^3^ d_max_ maximum degradation rate at T_P_ of each step_._^4^ T_0.05_ temperature at the 5% conversion degree. ^5^ T_max_ maxima decomposition temperature. ^6^ τ_1/2_ time at the 50% conversion degree.

**Table 4:** Cure parameters of benchmark compounds computed from DSC scans.

Composition	Φ (°C/min)	1	2	5	10	20
S_1_	c_max_ (min^−1^)	0.040	0.075	0.184	0.335	0.632
	T_0.01_^1^ (°C)	70	82	94	104	111
	T_p_^2^ (°C)	115	127	141	152	167
	T_0.999_^3^ (°C)	148	161	175	191	204
	ΔH ^4^ (J/g)	354	327	283	299	297
S_2_	c_max_ (min^−1^)	0.042	0.081	0.187	0.349	0.650
	T_0.01_ (°C)	64	65	90	99	106
	T_p_ (°C)	108	119	136	148	161
	T_0.999_ (°C)	147	153	168	182	197
	ΔH (J/g)	320	344	337	316	288
S_5_	c_max_ (min^−1^)	0.044	0.088	0.211	0.378	0.732
	T_0.01_ (°C)	53	67	78	87	95
	T_p_ (°C)	100	111	123	134	148
	T_0.999_ (°C)	147	150	158	168	179
	ΔH (J/g)	362	314	311	318	314

^1^ T_0.01_ temperature at 0.01 conversion degree (assumed as initial cure temperature). ^2^ T_p_ peak temperature. ^3^ T_0.999_ temperature at 0.999 conversion degree (assumed as final cure temperature). ^4^ ΔH overall reaction heat.

**Table 5:** Cure parameters M_X_ 100:87:X.

Composition	Φ(°C/min)	1	2	5	10	20
M_5_	c_max_ (min^−1^)	0.018	0.073	0.128	0.182	0.328
	T_0.1%_ (°C)	123	182	186	193	132
	T_p_ (°C)	189	215	234	244	244
	T_99.9%_ (°C)	221	263	284	306	325
	ΔH (J/g)	203	166	195	192	372
M_10_	c_max_ (min^−1^)	0.105	0.187	0.228	0.349	0.512
	T_0.1%_ (°C)	81	90	97	99	103
	T_p_ (°C)	125	136	144	148	157
	T_99.9%_ (°C)	154	168	176	182	188
	ΔH (J/g)	290	337	325	316	318

**Table 6:** Tensile properties of epoxy/eggshell compounds.

Composition	Young Modulus (GPa)	Tensile Strength (MPa)	Deformation (%)
S_5_	1.28 ± 0.26	18.13 ± 5.35	2.35 ± 0.60
E_10_	1.51 ± 0.20	22.80 ± 2.21	2.61 ± 0.03
EM_10_	1.40 ± 0.28	20.63 ± 4.88	2.33 ± 0.52
M_10_	1.12 ± 0.18	26.64 ± 4.81	3.17 ± 0.21

## Supplementary Materials

The following are available online at https://www.mdpi.com/1996-1944/12/9/1489/s1, Figure S1: TGA and DTG plots of the raw materials; Figure S2: DSC scans of S_X_ benchmark compounds at indicated heating rates. (Effect of heating rates); Figure S3: DSC scans of S_X_ benchmark compounds at indicated heating rates. (Effect of DEH 35 content); Figure S4: Degree of conversion of S_X_ at indicated heating rates; Figure S5: Degree of conversion of S_X_ at indicated heating rates. (Effect of DEH 35 content); Figure S6: Conversion rate of S_X_ compounds at indicated heating rates; Figure S7: Conversion rate of S_X_ with the same heating rate at different compositions; Figure S8: DSC scans of E_X_/EM_X_/M_X_, at indicated heating rates; Figure S9: DSC scans of E_X_/EM_X_/M_X_, at indicated heating rates; Figure S10: Degree of conversion of M_X_ at indicated heating rates; Figure S11: Conversion rate of M_X_ at indicated heating rates.
