# Supplementary material for: Optimization of Epoxy Resin: An Investigation of Eggshell as a Synergic Filler
Source: Materials (Basel). 2019 May 8;12(9):1489. doi: 10.3390/ma12091489 (PMC6540584; doi:10.3390/ma12091489)
Supplement: Supplementary file 1 [file materials-12-01489-s001.pdf]

# Supplementary Materials: Optimization of Epoxy Resin: An Investigation of Eggshell as a Synergic Filler

**José William de Lima Souza [1], Nichollas Guimarães Jaques [1,2], Matthias Popp [3], Jana Kolbe [3], Marcus Vinícius Lia Fook [1] and Renate Maria Ramos Wellen [1,2,*]**

## 1. Thermogravimetry Analyses—TGA

**Methodology:** Sample tests were analyzed in TGA Pyris-1 from Perkin Elmer (Waltham, MA, USA). Samples with approximately 5 mg were heated from ambient temperature ($T_{amb}$) to 900 °C using a heating rate of 10 °C/min under synthetic air with gas flow of 20 mL/min.

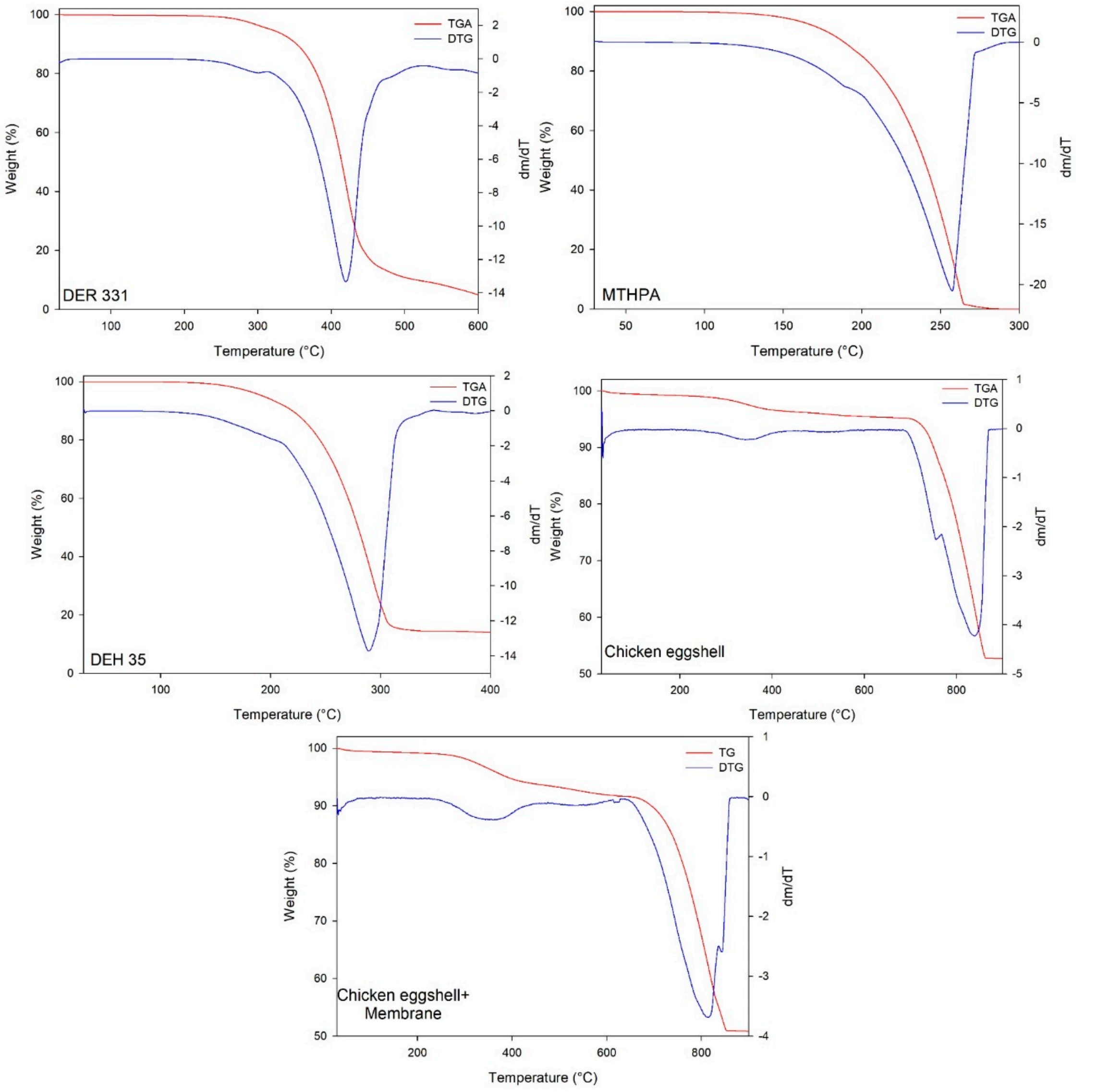


**Figure S1.** TGA and DTG plots of the raw materials.

## 2. Differential Scanning Calorimetry Measurements—DSC

**Methodology**: The equipment used was a DSC Q20 from TA Instruments (New Castle, DE, USA), samples with approximately weight of 5mg were tested in standard closed aluminum pan,

under a nitrogen gas flow of 50 mL/min. The samples were heated from Ambient Temperature (Tamb) to 400 °C, at the heating rates: 1, 2, 5, 10 and 20 °C/min.

**Data Processing and Graphic Plots:** Raw data acquired from the instrument software were analyzed using a custom software INTEGRAL1B, and then plotted using Sigmaplot.

The raw data acquired from the instrument are:

Time (t) in minutes;

Temperature (T) in °C;

Heat flow in mW.

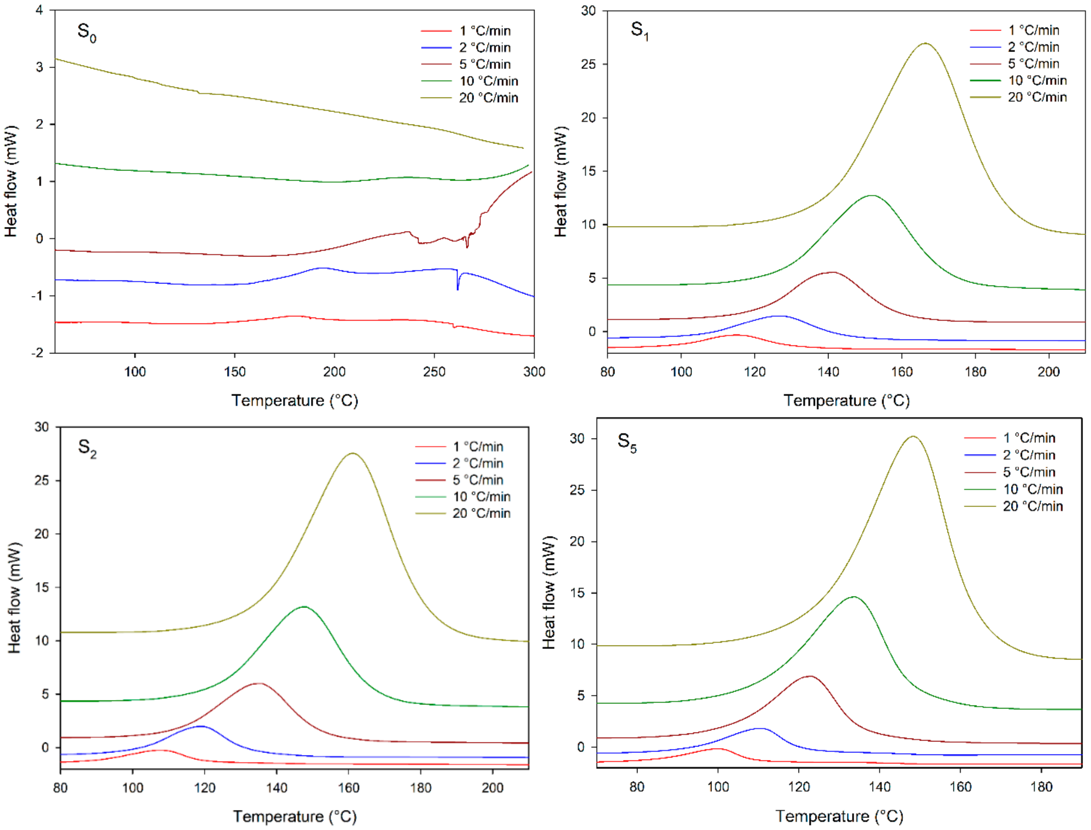


**Figure S2.** DSC scans of Sx benchmark compounds at indicated heating rates. (Effect of heating rates).

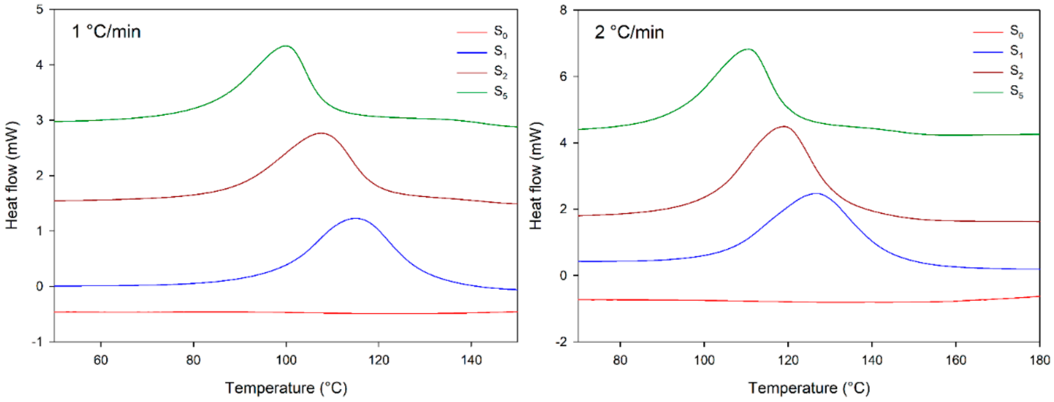

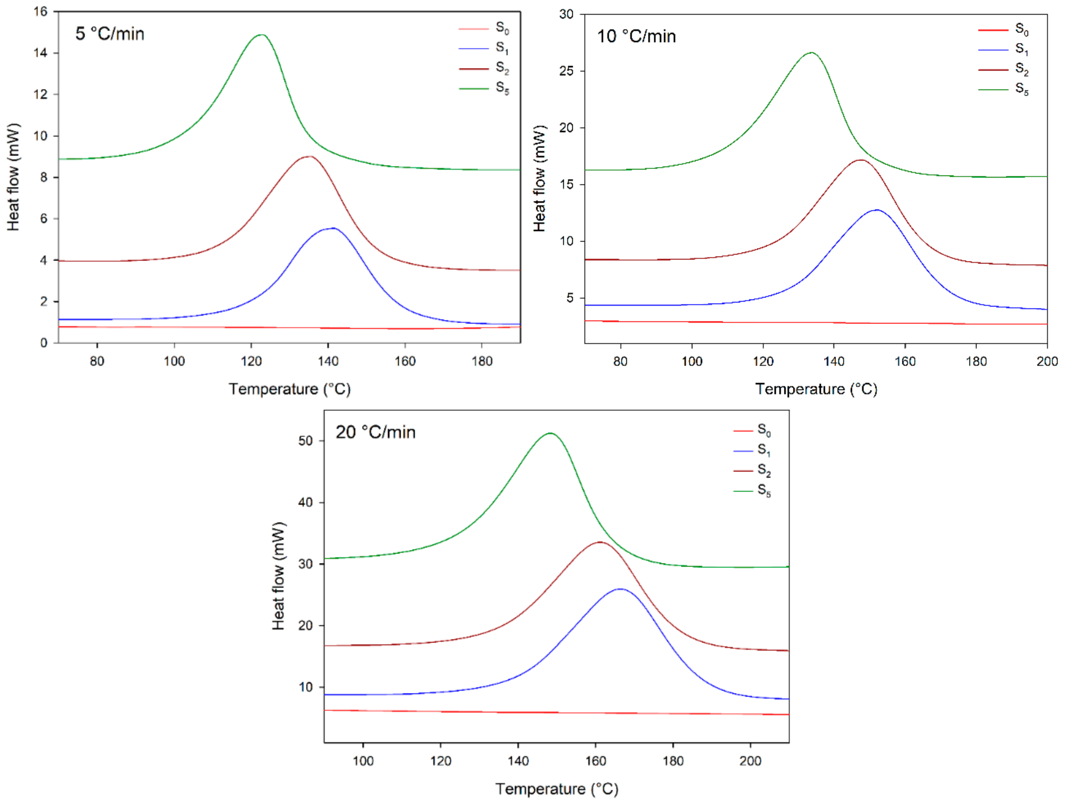


**Figure S3.** DSC scans of Sx benchmark compounds at indicated heating rates. (Effect of DEH 35 content).

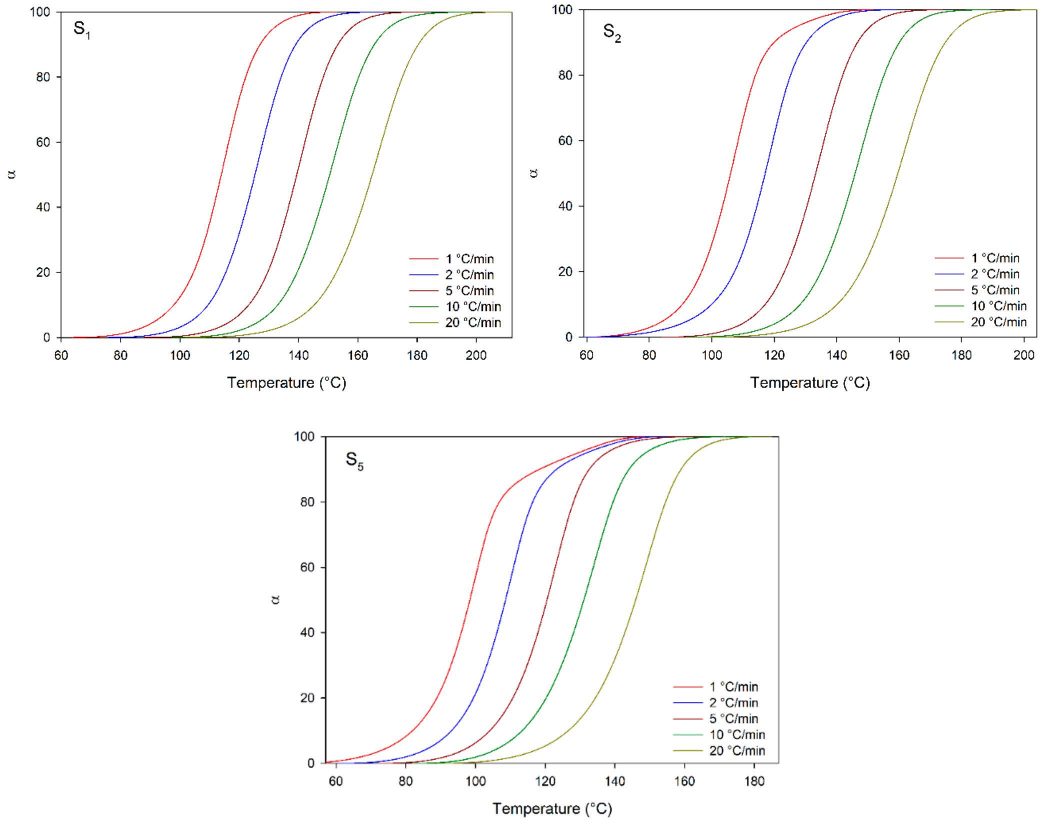


**Figure S4.** Degree of conversion of Sx at indicated heating rates.

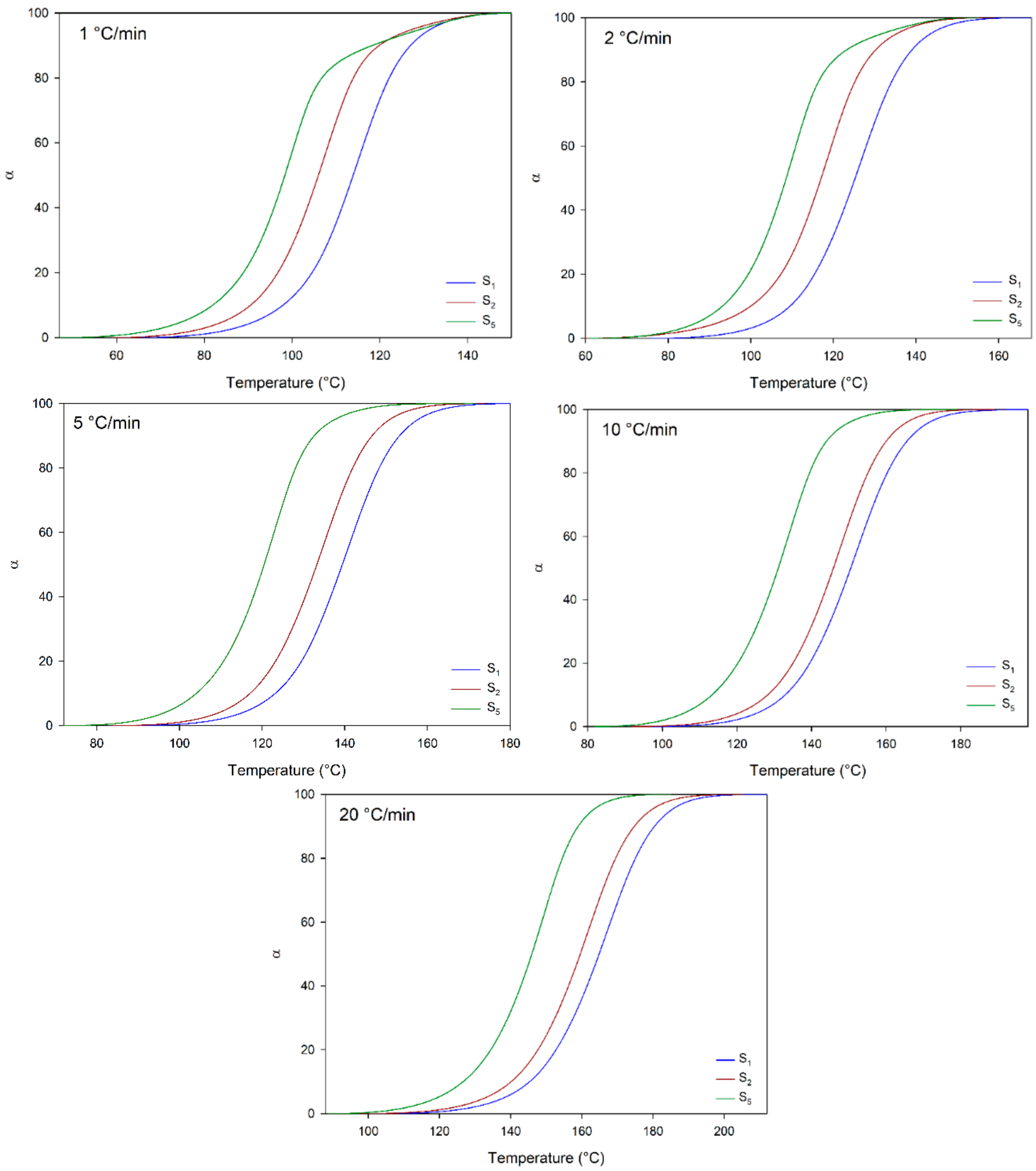


**Figure S5.** Degree of conversion of Sx at indicated heating rates. (Effect of DEH 35 content).

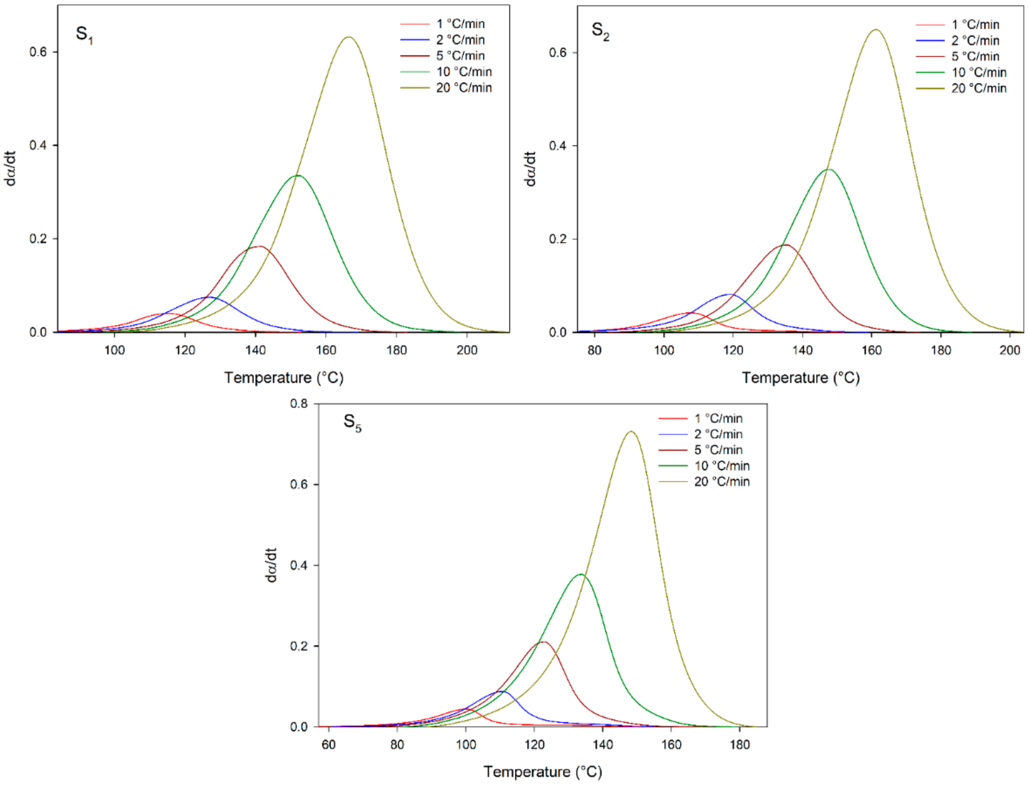


**Figure S6.** Conversion rate of Sx compounds at indicated heating rates.

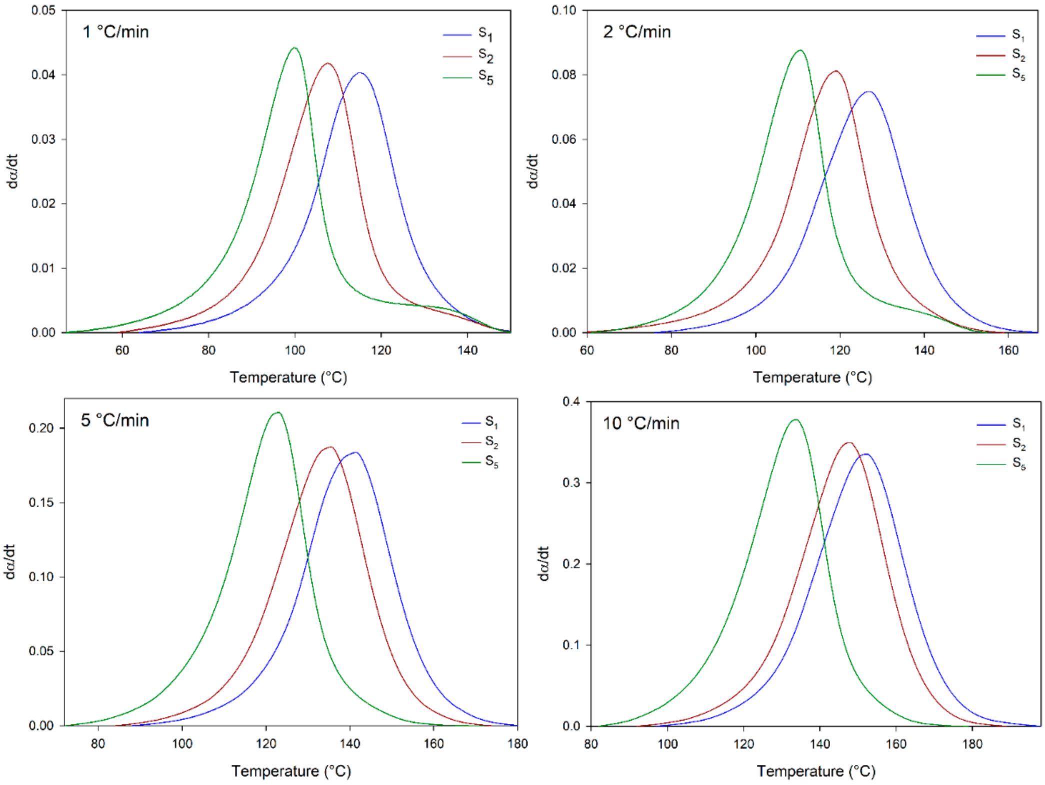

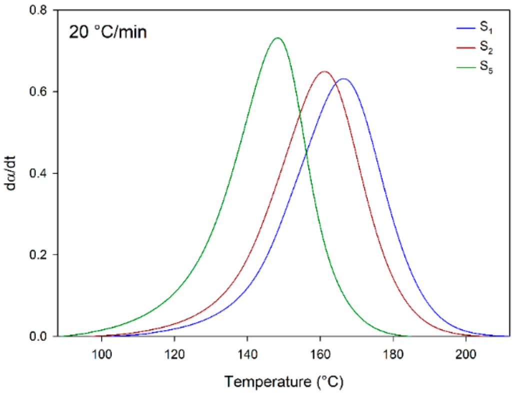


**Figure S7.** Conversion rate of Sx with the same heating rate at different compositions.

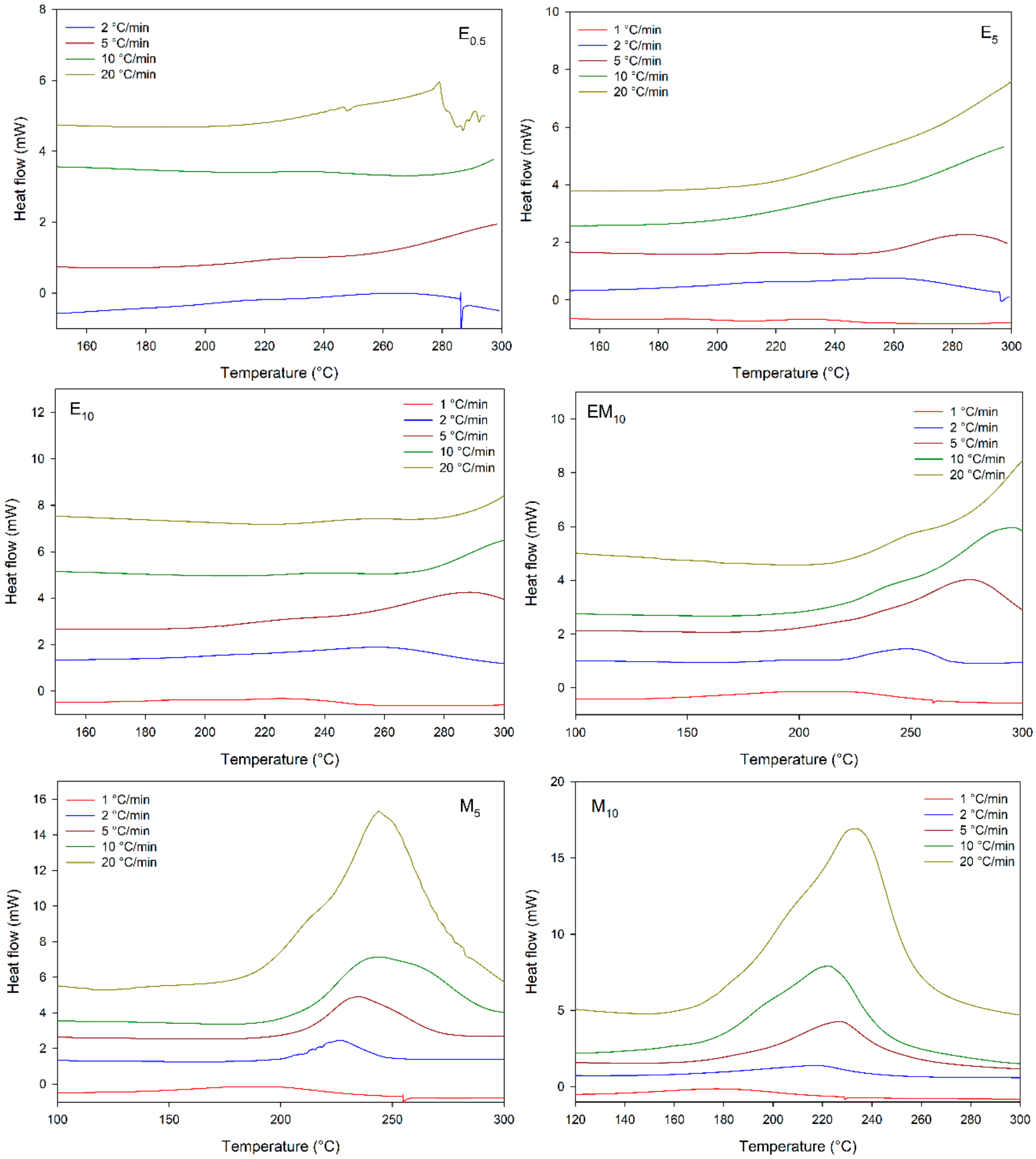


**Figure S8.** DSC scans of Ex/EMx/Mx, at indicated heating rates.

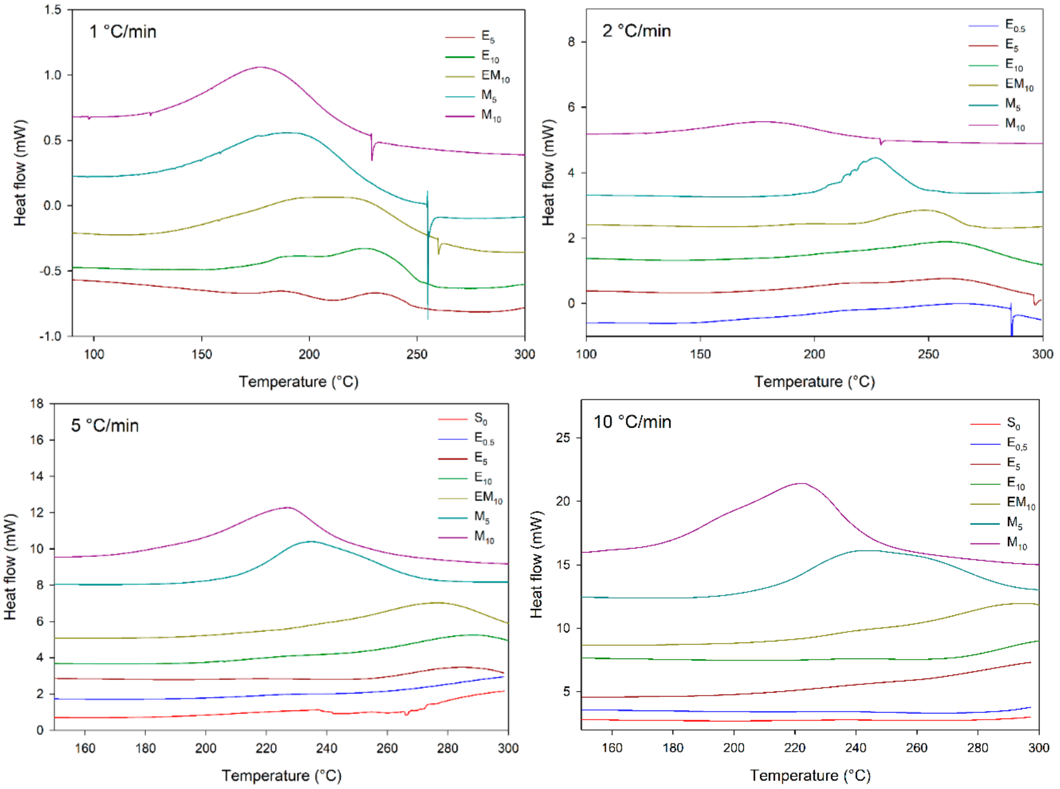


**Figure S9.** DSC scans of Ex/EMx/Mx, at indicated heating rates.

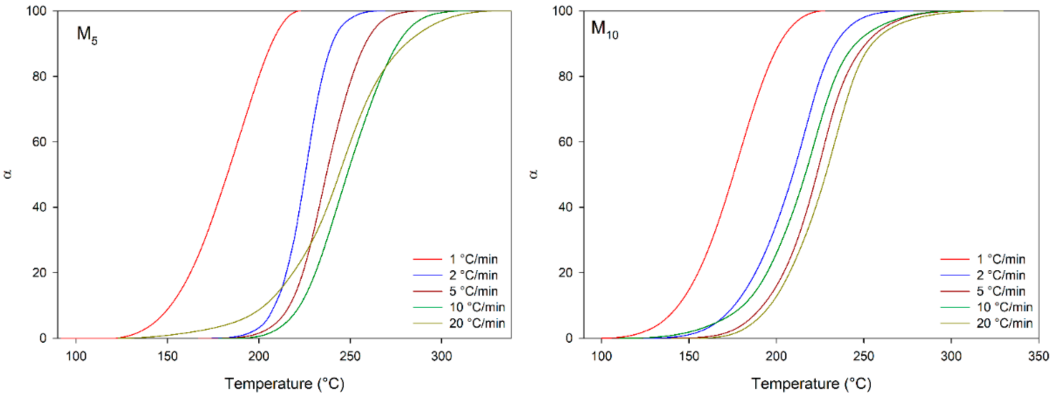


**Figure S10.** Degree of conversion of Mx at indicated heating rates.

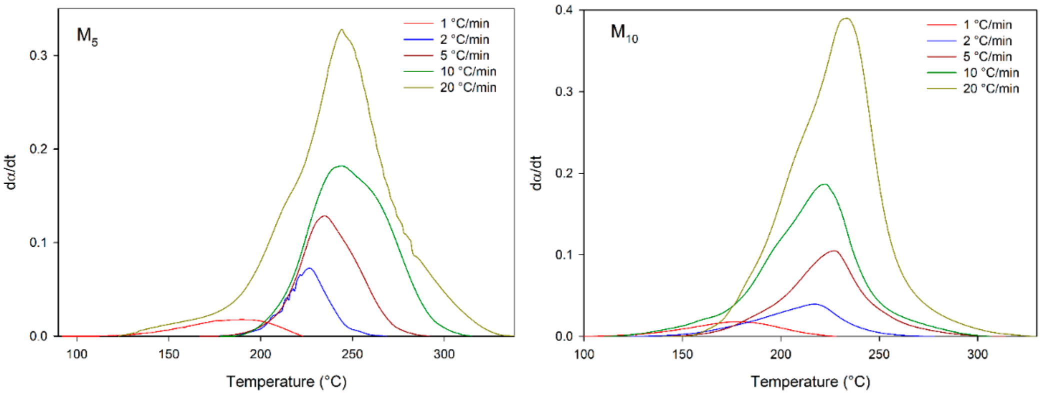


**Figure S11.** Conversion rate of Mx at indicated heating rates.
